# Association between maternal antidepressant use during pregnancy and autism spectrum disorder: an updated meta-analysis

**DOI:** 10.1186/s13229-018-0207-7

**Published:** 2018-03-27

**Authors:** Xi-Hong Zhou, Yong-Jiang Li, Jian-Jun Ou, Ya-Min Li

**Affiliations:** 1 Clinical Nursing Teaching and Research Section, The Second Xiangya Hospital, Central South University, Changsha, 410011 Hunan China; 2 Department of Pharmacy, The Second Xiangya Hospital, Central South University, Changsha, 410011 Hunan China; 3 Institute of Clinical Pharmacy, Central South University, Changsha, 410011 Hunan China; 4 Mental Health Institute, The Second Xiangya Hospital, Central South University, Changsha, 410011 Hunan China

**Keywords:** Antidepressant exposure, Autism spectrum disorder, Offspring, Meta-analysis

## Abstract

Studies have investigated the risk of autism spectrum disorder (ASD) in children exposed in utero to antidepressant, with inconsistent results. Given the substantial public health implications on this topic, here, we presented an updated meta-analysis of the association between maternal antidepressant use during pregnancy and ASD. Cochrane Library, EMBASE, PsycINFO, and PubMed databases were systematically searched. A random effects model was used to pool the adjusted relative risk (RR) for cohort studies and the adjusted odds ratio (OR) for case-control studies as well as their corresponding 95% confidence intervals (CIs). Meta-analysis restricted to sibling studies was also conducted. Publication bias was systematically assessed. Fourteen studies were identified (eight cohort studies and six case-control studies). Pooled adjusted RR for cohort studies (*n* = 2,839,980) was 1.13 (0.93–1.39) showed a non-significant association; while two studies were potentially missing from the test of publication bias, filled estimates also showed a non-significant association (filled RR 0.97, 95% CI 0.79–1.19). Pooled OR was 1.51 (1.15–1.99) for case-control studies (*n* = 117,737) showed a significant association; two studies were potentially missing; however, the filled estimates suggested a non-significant association (filled OR 1.26, 95% CI 0.98–1.62). Analyses restricted to sibling studies also showed a non-significant association (RR 0.99, 95% CI 0.81–1.22). In summary, we did not evidence a significant association between maternal antidepressant use during pregnancy and ASD.

**Electronic supplementary material:**

The online version of this article (10.1186/s13229-018-0207-7) contains supplementary material, which is available to authorized users.

## Introduction

Nearly 15% of pregnant women have depression [1]. Untreated depression during pregnancy may lead to significant obstetrical and developmental issues, including gestational diabetes, hypertension, preeclampsia, postnatal complications and suicidal ideation on mothers, hyperactivity and irregular heart rate on developing fetus, increased rates of premature deaths on newborns as well as internalizing and externalizing problems on children [2]. Therefore, treatment of depression during pregnancy is of great significance for both mothers and children.

Antidepressants are widely used medications for the treatment of prenatal depression, and selective serotonin reuptake inhibitors (SSRIs) were the most commonly prescribed. With the considerably increased prevalence of antidepressant use during pregnancy, confusion continued regarding the appropriate use of antidepressants during this critical period in consideration of maternal and fetal outcomes.

Previous meta-analyses have evaluated the association between antidepressant use during pregnancy and the risk of autism spectrum disorders (ASDs) in children [3–9], all reported a significant association. However, those meta-analyses concluded a positive association did not cover several important eligible studies, including the cohort study by Malm et al. [10] and other four newly published large-scale cohort studies [11–14]. Further, publication bias has not been comprehensively examined in previous meta-analyses.

In recent two large-scale cohort studies that aimed to further disentangled the associations of maternal depression, antidepressant exposure during pregnancy, and ASD risk in offspring [11, 12], researchers reported that their data analysis of large, population-based sample with adequate statistical power showed that maternal antidepressant exposure was not associated with ASD. Also, they emphasized the fact that regardless of antidepressant treatment, maternal depression without treatment remains at increased risk for developmental disorders in children.

As the use of antidepressants is likely to be remained widely for the treatment of depression during pregnancy, the precise estimation of the potential risk has substantial public health implications. Hence, we presented an updated meta-analysis based on current evidence to first evaluate whether maternal antidepressant use during pregnancy is a risk factor for ASD and to second comprehensively assess the publication bias.

## Method

### Search strategies and eligibility criteria

A systematic literature search of Cochrane Library, EMBASE, PsycINFO, and PubMed databases was conducted in July 2017, using the combination of Medical Subject Headings and relevant keywords as follows: autism/autistic/asperger/autism spectrum disorder/ASD and antidepressant/antidepressive agents/selective serotonin uptake inhibitor/SSRI/ and maternal/pregnancy/fetal/children/offspring. Reference lists from articles as well as meta-analyses identified by the search were also screened to identify additional studies. Two reviewers performed the searches, screened title/abstracts, and reviewed full text independently. Articles were included if they reported original case-control or cohort studies that investigated the risk of ASD in children exposed in utero to antidepressant; articles were excluded if they reported cases or case series.

### Data extraction

The baseline characteristics of included studies, including the first author’s name, publication year, study design, data source, sample size, adjusted covariates, and main findings (adjusted estimates and their corresponding 95% CIs), were reviewed and extracted.

### Quality assessment

The improved Newcastle-Ottawa Scale [15] was used for quality assessment of included studies. Three domains were evaluated, including selection, comparability, and exposure. Each satisfactory answer was assigned one “star,” and studies with six or more stars were considered of high quality. Two authors independently assessed study quality. Consensus was reached by discussion with the primary author.

### Data analysis

We first analyzed the overall impact of any antidepressant exposure and second analyzed the specific impact of SSRIs to investigate whether the association between SSRI exposure and ASD would be influenced by other antidepressants. After careful review of the reported data from the original publications, the exposure period of prepregnancy, first trimester, second and/or third trimester, and the whole pregnancy were investigated in cohort studies. While for case-control studies, the exposure period of prepregnancy, first trimester, second trimester, third trimester, and the whole pregnancy were investigated. Therefore, in consideration of methodological heterogeneity between cohort studies and case-control studies, data from cohort studies and case-control studies were separately pooled.

A random effects model was used for meta-analyses. Heterogeneity was evaluated with the *I*^2^ statistic, *P* value, and tau value. Funnel plots were produced to evaluate publication bias. If potential publication bias was observed by visually examining the asymmetry of the funnel plots, then the “trim and fill” method would be applied to adjust the funnel plot and further to recalculate the pooled estimates. In addition, to test the robustness of the pooled estimates, sensitivity analyses were performed for studies with overlapping data sources by deleting each study and rerunning meta-analysis. Confounding by indication was first addressed by excluding studies that did not include maternal psychiatric conditions as covariates, and second addressed by performing meta-analysis on cohort studies employing more rigorous controls such as sibling controls or controls of children exposed to maternal psychiatric disorder but no antidepressant use during pregnancy. All data analyses were performed using Stata statistical software (version 12.0; Stata Corporation, College Station, TX, USA).

## Results

### Study selection and baseline characteristics

Flow diagram of the study selection process was presented in Additional file 1: Figure S1. Briefly, 213 records were retrieved from our systematic literature search. After removing duplicates and irrelevant titles, 23 articles remained for full-text screening. Eight studies that did not report original investigations and one study did not address the outcome of interest were excluded. Finally, 14 studies met the eligibility criteria were included [10–14, 16–24]. Baseline characteristics of the included studies were presented in Additional file 2: Table S1. Three out of six case-control studies, two out of eight cohort studies reported a positive association. Two case-control studies did not include maternal psychiatric disorders as covariates in their multivariate analysis [16, 21]. In quality assessment, NOS scores for all included studies were high and all were rated as high quality (Additional file 3: Table S2).

### Meta-analysis

**Fig. 1:**
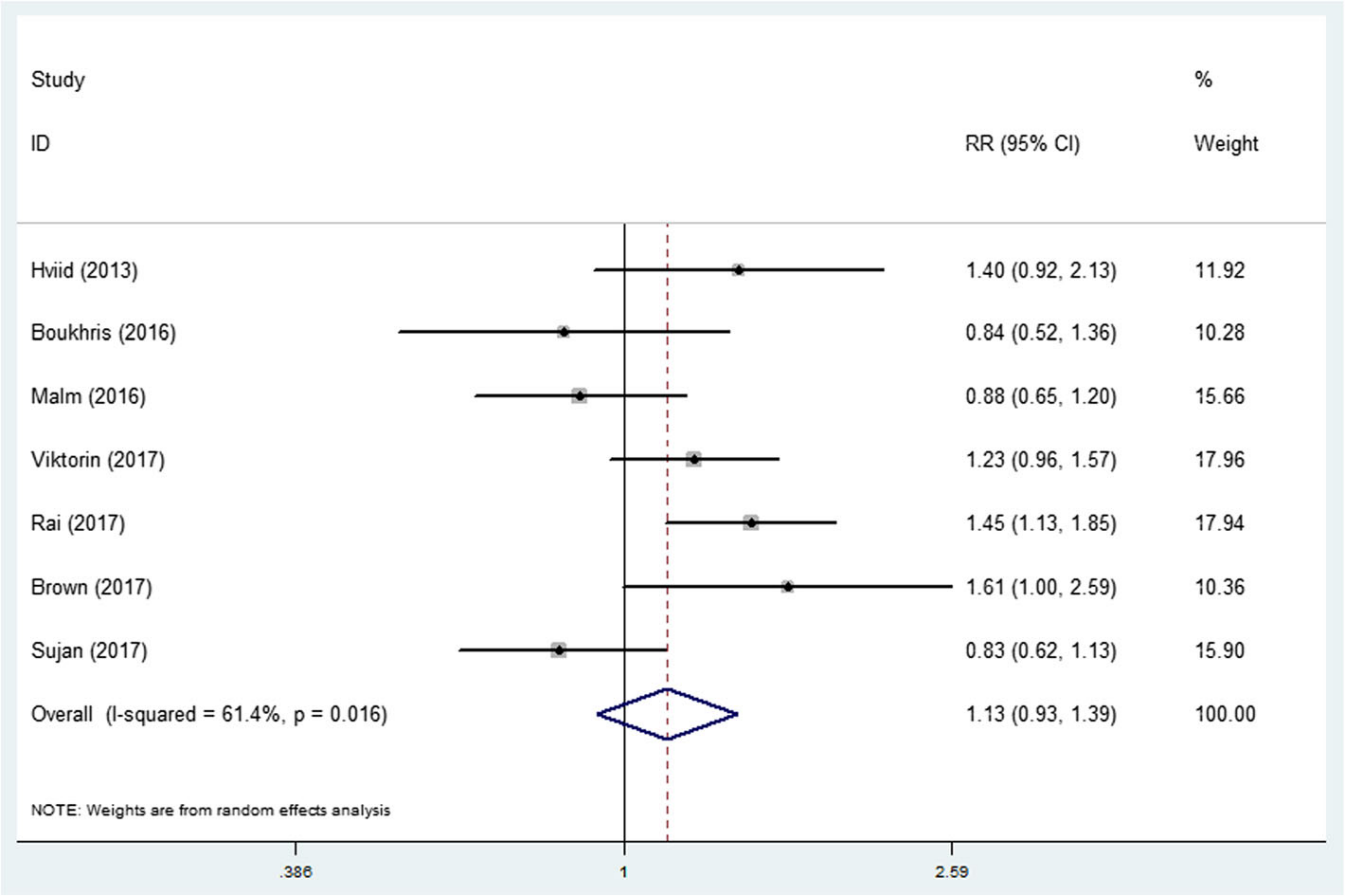
Forest plot of the meta-analysis of cohort studies on the effects of prenatal antidepressant exposure and autism spectrum disorder

**Fig. 2:**
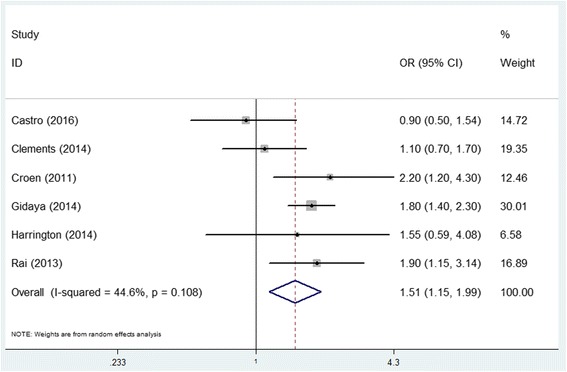
Forest plot of the meta-analysis of case-control studies on the effects of prenatal antidepressant exposure and autism spectrum disorder

Pooled adjusted RR for cohort studies (*n* = 2,839,980) was 1.13 (95% CI 0.93–1.39) with moderate between-study heterogeneity (*I*^2^ = 61.4%, *P* = 0.016) showed a non-significant association between prenatal antidepressants exposure and ASD (Fig. 1). Pooled adjusted OR for case-control studies (*n* = 117,737) was 1.51 (95% CI 1.15–1.99) with moderate between-study heterogeneity (*I*^2^ = 44.6%, *P* = 0.108) showed a statistically significant association (Fig. 2).

Results of meta-analyses stratified by pregnancy period and medication were summarized in Table 1. A non-significant association was found for pooled RR for cohort studies, while the pooled ORs for case-control studies showed a significant association. As for different trimesters, the pooled results did not alter for both cohort studies and case-control studies. The effect of SSRI was slightly larger than the combined effect of any antidepressant. Although there were two cohort studies with overlapping data sources [17, 19], the sample was not duplicated as data were not pooled in any subgroup in our analysis. As for confounding by indication, the pooled OR was changed to 1.41 (95% CI 1.00–1.99) after excluding the two case-control studies that did not include maternal psychiatric disorders as covariates in their multivariate analysis (Additional file 4: Figure S2). The pooled RR for cohort studies with sibling controls or controls of children exposed to maternal psychiatric disorder, but no antidepressant use during pregnancy was 0.99 (95% CI 0.81–1.22), with substantially decreased heterogeneity between studies (*I*^2^ = 9.0%, *P* = 0.355) (Additional file 5: Figure S3).

**Table 1:** Summary of the meta-analyses performed on fetal antidepressant exposure during pregnancy and autism spectrum disorders

			Overall effect			Heterogeneity		
	Medication	No. of studies	OR/HR (95% CI)	*Z*	*P* value	*I*^2^ (%)	*P*	Tau
Cohort studies								
Pregnancy	Any antidepressant	7	1.13 (0.93–1.39)	1.22	0.224	61.4	0.016	0.0437
	Any SSRI	3	1.22 (0.83–1.79)	0.99	0.320	64.7	0.059	0.0749
First trimester	Any antidepressant	5	1.01 (0.82–1.24)	0.06	0.953	31.9	0.209	0.0174
	Any SSRI	3	1.04 (0.81–1.34)	0.32	0.747	42.7	0.155	0.027
Second and/or third trimester	Any antidepressant	4	1.35 (0.96–1.90)	1.75	0.080	46.4	0.133	0.0552
	Any SSRI	3	1.43 (0.84–2.42)	1.32	0.187	71.6	0.030	0.1549
Prepregnancy	Any antidepressant	2	1.26 (0.91–1.74)	1.40	0.160	67.1	0.081	0.0365
	Any SSRI	1	1.46 (1.17–1.81)					
Case-control studies								
Pregnancy	Any antidepressant	6	1.51 (1.15–1.99)	3.00	0.003	44.6	0.108	0.0478
	Any SSRI	4	1.81 (1.46–2.23)	5.50	< 0.001	0.0	0.908	< 0.0001
First trimester	Any antidepressant	5	1.69 (1.17–2.44)	2.81	0.005	46.3	0.114	0.0756
	Any SSRI	2	2.08 (1.54–2.81)	4.78	< 0.001	7.2	0.340	0.0098
Second trimester	Any antidepressant	5	1.62 (1.22–2.16)	3.31	0.001	8.6	0.358	0.0107
	Any SSRI	3	1.94 (1.41–2.68)	4.04	< 0.001	0.0	0.413	< 0.0001
Third trimester	Any antidepressant	5	1.47 (0.88–2.44)	1.48	0.140	60.0	0.040	0.1843
	Any SSRI	3	2.32 (1.63–3.30)	4.68	< 0.001	0.0	0.515	< 0.0001
Prepregnancy	Any antidepressant	4	1.70 (1.43–2.02)	6.04	< 0.001	0.0	0.897	< 0.0001
	Any SSRI	2	1.81 (1.43–2.29)	4.92	< 0.001	0.0	0.896	< 0.0001

### Publication bias

**Fig. 3:**
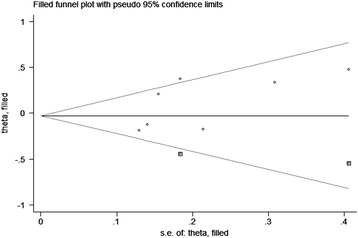
Filled funnel plot of included cohort studies showing number of potential missing studies

**Fig. 4:**
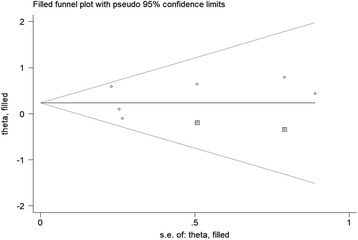
Filled funnel plot of included case-control studies showing number of potential missing studies

Although the number of included studies was limited, funnel plots for cohort studies and case-control studies were produced separately for assessment of publication bias, and all were obviously asymmetry, suggesting potential publication bias (Additional file 6: Figure S4; Additional file 7: Figure S5). Therefore, “trim and fill” analysis was performed to estimate the number of missing studies. As a result, for both cohort studies and case-control studies, two potential missing studies were detected by adjusting the funnel plot (Figs. 3 and 4). However, both filled estimates demonstrated a non-significant association (filled RR for cohort studies 0.97, 95% CI 0.79–1.19; filled OR for case-control studies 1.26, 95% CI 0.98–1.62).

## Discussion

Our meta-analysis is based on published observational studies pertaining to ASD risk of in utero exposure to antidepressant. Data from cohort studies and case-control studies were synthesized separately due to inconsistent study design, which is a significant source of heterogeneity. Results from our meta-analysis showed that no association was found for cohort studies; while the pooled OR for case-control studies showed a significant association, the filled OR was dramatically changed and the association was changed accordingly to be non-significant.

Our systematic literature search identified seven published meta-analyses on this topic [3–9], all reported a significant association from their data analysis. However, those meta-analyses did not include all eligible original investigations, especially for several newly published large scale cohort studies [10–14], and thus concluded a positive association. Besides, publication bias was not systematically evaluated in previous meta-analyses due to insufficient number of eligible studies. Hence, the weight of case-control studies was very high and may thus lead to a false-positive result and a misleading conclusion. Most cohort studies did not replicate such a significant association reported in most case-control studies. Compared with cohort studies, case-control studies were generally retrospective and unable to control for important confounding factors (i.e., indication) thus less likely to evidence a causal association. In addition, the pooled sample size of case-control studies (*n* = 117,737) was much smaller than that of cohort studies (*n* = 2,839,980).

The combined effect of confounding by indication and publication bias may explain the difference between cohort studies and case-control studies. After excluding the two case-control studies that did not include maternal psychiatric disorders as covariates [16, 21], the pooled estimate was significantly changed (OR 1.41, 95% CI 1.00–1.99). Results of meta-analysis on cohort studies with sibling controls or controls of children exposed to maternal psychiatric disorder but no antidepressant use during pregnancy showed a non-significant association (RR 0.99, 95% CI 0.81–1.22) with less heterogeneity (*I*^2^ = 9.0%, *P* = 0.355) suggested that the residual confounding of maternal psychiatric disorders might be an important source of heterogeneity. More importantly, in our test of publication bias, two potential missing studies were detected by “trim and fill” method for both cohort studies and case-control studies, and the filled estimates for cohort studies still suggested a non-significant association between maternal antidepressant use and ASD, but the filled estimates for case-control studies dramatically changed and demonstrated a non-significant association.

Findings from studies should be treated with caution in consideration of study limitations and in case of false-positive results. Indeed, medication use during pregnancy has been concern of clinicians and mother-to-be. Nevertheless, the devastating effects of untreated depression during pregnancy on the mothers as well as on the developing children have been widely studied and reported [2]. More seriously, prenatal depression is characterized by a relatively high frequency of somatic symptoms and suicide ideation that may lead to grave consequences [25].

The limitation for this meta-analysis is the unexplored validity of the test of publication bias as it contributed significantly to our conclusions. The “trim and fill” method for testing and adjusting publication bias is funnel-plot-based. The number of missing studies and their effect sizes were estimated and calculated rather than retrieved. Therefore, the filled studies may not match reality in any close way and the filled estimates may not reflect the precision.

In conclusion, this meta-analysis based on current best evidence and conducted with an appropriate approach quantitatively concluded that maternal antidepressant exposure may not be associated with ASD. In this case, we do not suggest pregnant women diagnosed with severe depression discontinuing their antidepressant use for fear of an uncertain risk. Nevertheless, despite the non-significant association we currently concluded, information about doses of antidepressant exposure and severity of ASD symptoms is less, and the absence of population-level, individual-specific data on depression, and ASD warranted further investigations.

## Additional files

Additional file 1: **Figure S1.** PRISMA diagram for the study selection process. (TIFF 306 kb)

Additional file 2: **Table S1.** Baseline characteristics and main findings of included studies in the meta-analysis. (DOCX 22 kb)

Additional file 3: **Table S2.** Quality assessment of the included studies by the improved Newcastle–Ottawa Scale. (DOCX 16 kb)

Additional file 4: **Figure S2.** Forest plot for case-control studies adjusted for maternal psychiatric disorders in their multivariate analysis. (TIFF 42 kb)

Additional file 5: **Figure S3.** Forest plot for cohort studies with sibling controls or controls of children exposed to maternal psychiatric disorder but no antidepressant use during pregnancy. (TIFF 43 kb)

Additional file 6: **Figure S4.** Funnel plot of included cohort studies. (TIFF 40 kb)

Additional file 7: **Figure S5.** Funnel plot of included case-control studies. (TIFF 359 kb)

## Abbreviations

ASD: Autism spectrum disorder
CI: Confidence interval
OR: Odds ratio
RR: Relative risk
