# Supplementary figures and images for: Association between maternal antidepressant use during pregnancy and autism spectrum disorder: an updated meta-analysis
Source: Mol Autism. 2018 Mar 27;9:21. doi: 10.1186/s13229-018-0207-7 (PMC5870683; doi:10.1186/s13229-018-0207-7)

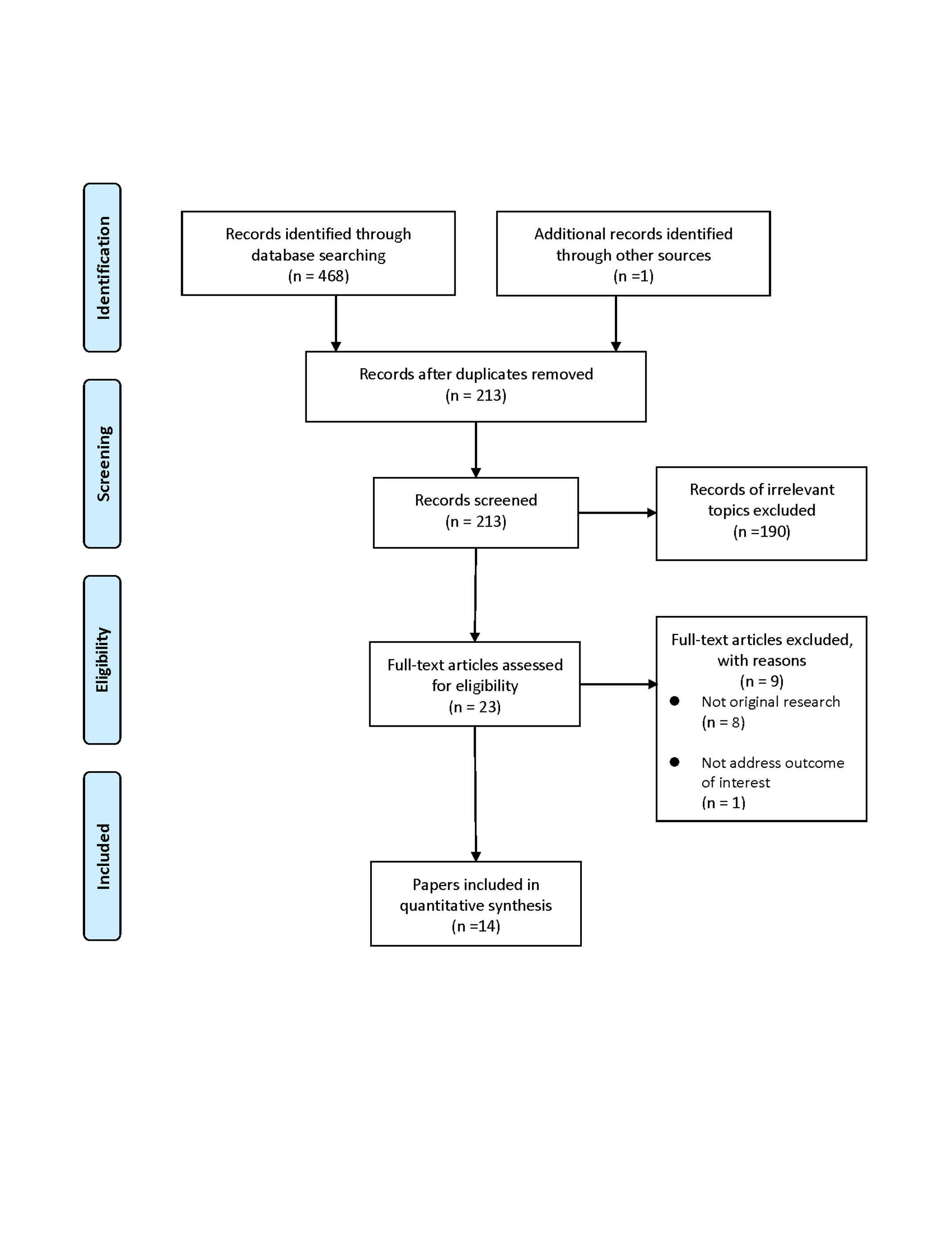

Supplement: Supplementary file 1 — Figure S1. PRISMA diagram for the study selection process. (TIFF 306 kb) [file 13229_2018_207_MOESM1_ESM.tif]

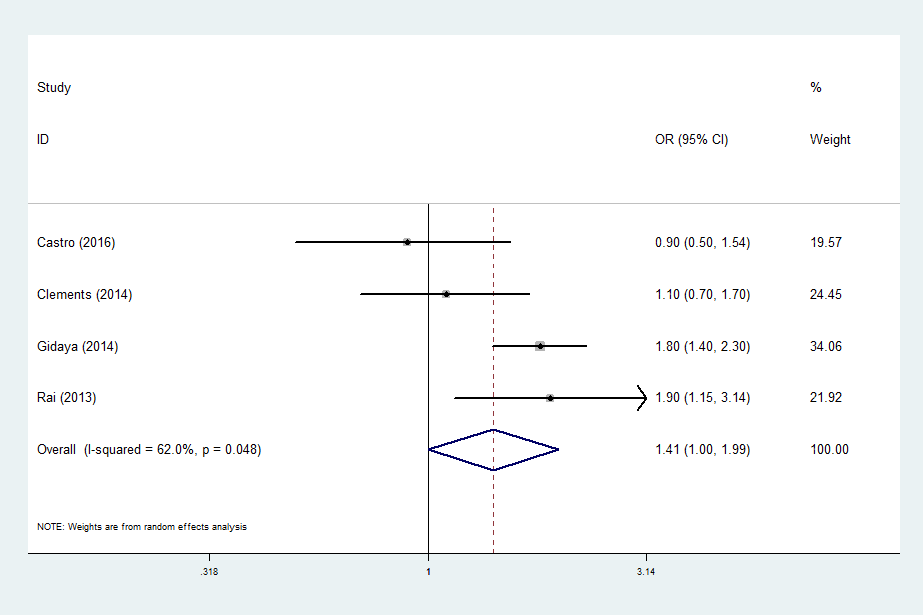

Supplement: Supplementary file 4 — Figure S2. Forest plot for case-control studies adjusted for maternal psychiatric disorders in their multivariate analysis. (TIFF 42 kb) [file 13229_2018_207_MOESM4_ESM.tif]

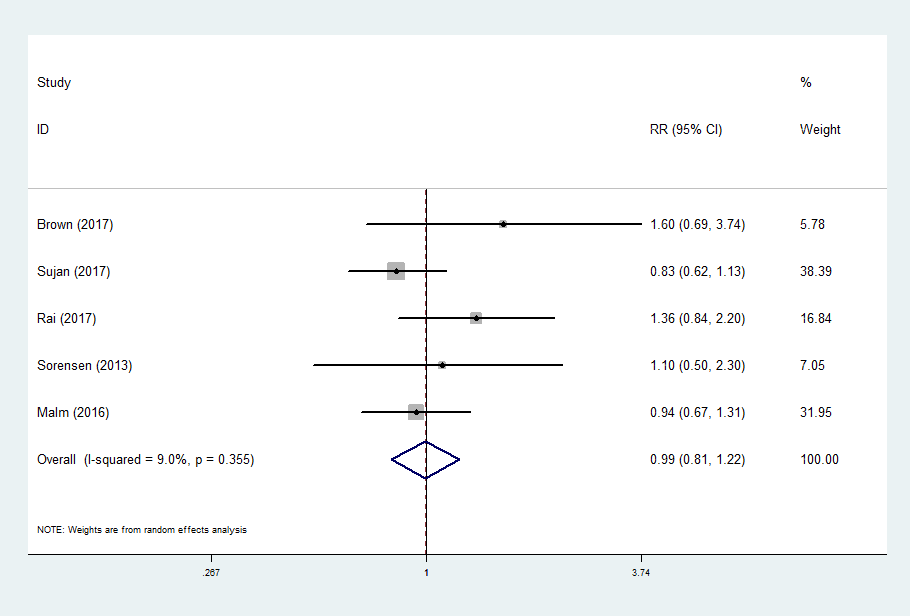

Supplement: Supplementary file 5 — Figure S3. Forest plot for cohort studies with sibling controls or controls of children exposed to maternal psychiatric disorder but no antidepressant use during pregnancy. (TIFF 43 kb) [file 13229_2018_207_MOESM5_ESM.tif]

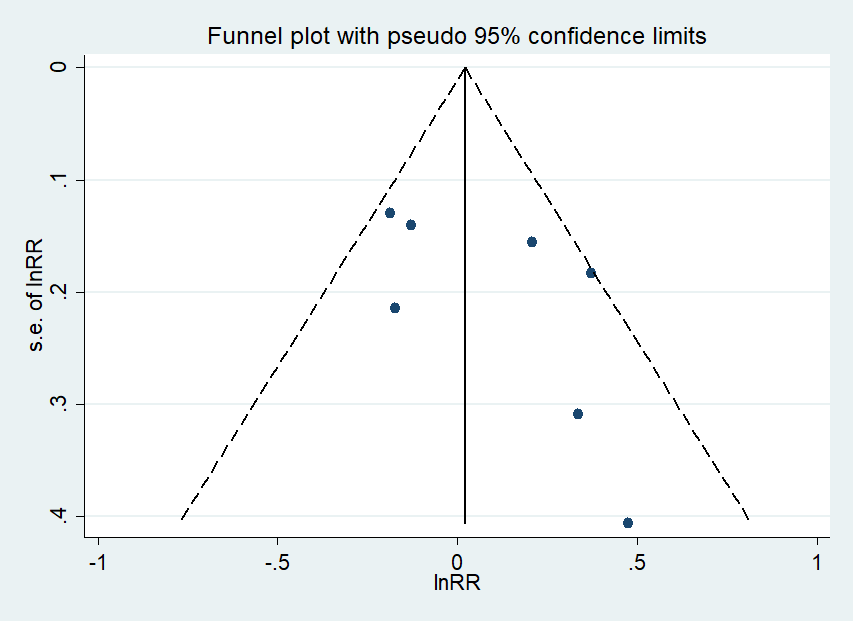

Supplement: Supplementary file 6 — Figure S4. Funnel plot of included cohort studies. (TIFF 40 kb) [file 13229_2018_207_MOESM6_ESM.tif]

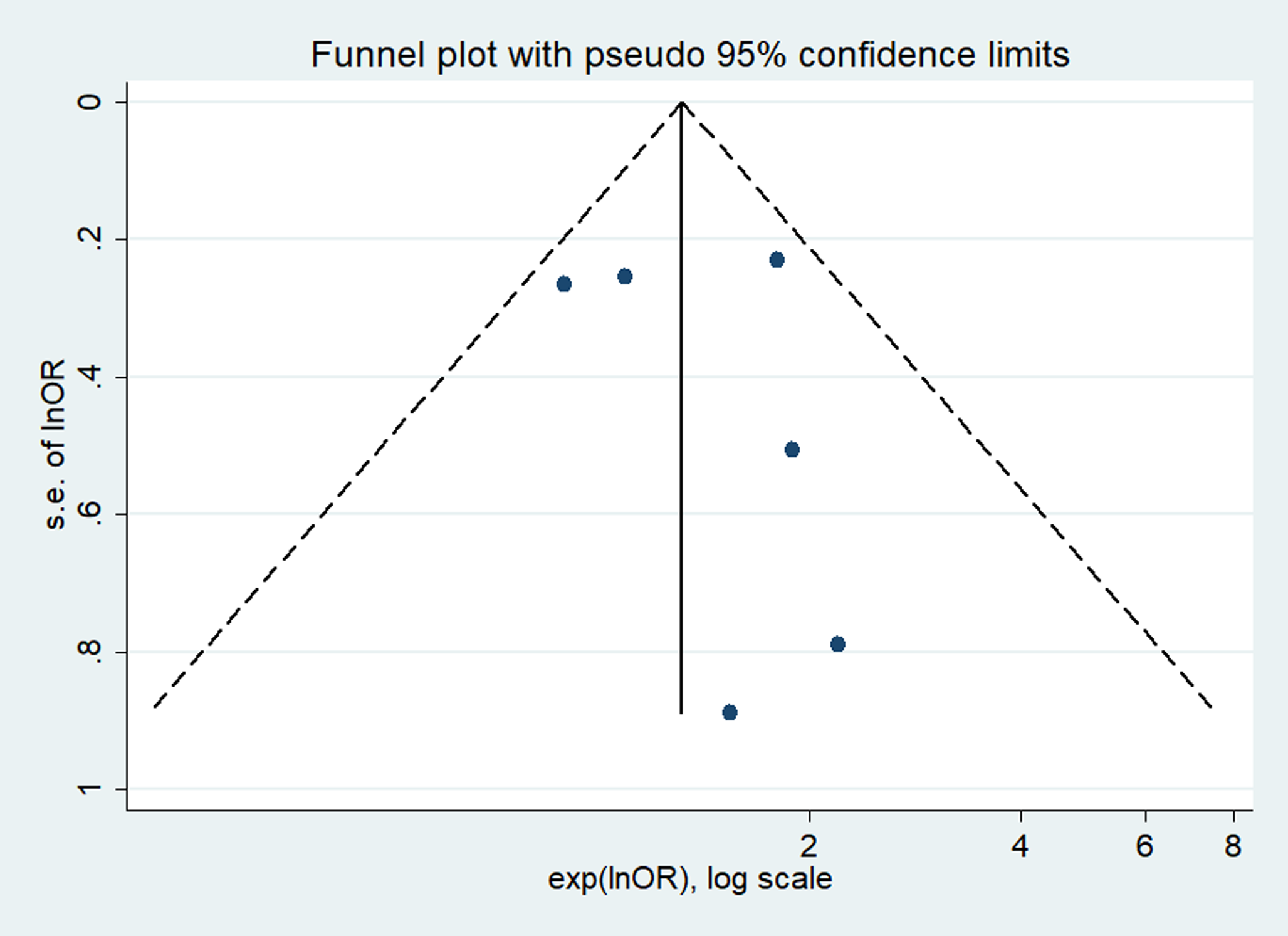

Supplement: Supplementary file 7 — Figure S5. Funnel plot of included case-control studies. (TIFF 359 kb) [file 13229_2018_207_MOESM7_ESM.tif]
