# Supplementary material for: Association between maternal antidepressant use during pregnancy and autism spectrum disorder: an updated meta-analysis
Source: Mol Autism. 2018 Mar 27;9:21. doi: 10.1186/s13229-018-0207-7 (PMC5870683; doi:10.1186/s13229-018-0207-7)
Supplement: Supplementary file 2 — Table S1. Baseline characteristics and main findings of included studies in the meta-analysis. (DOCX 22 kb) [file 13229_2018_207_MOESM2_ESM.docx]

Table S1. Baseline characteristics and main findings of included studies in the meta-analysis

| Study | Data source | Sample size | Adjusted covariates | Main findings  (Adjusted estimates with 95% CI) |
| --- | --- | --- | --- | --- |
| Cohort Studies | | | | |
| Malm et al.  (2016) | The National Medical Birth Register | Exposed 15,729  Unexposed 9,651 | Sex; maternal age; socioeconomic status; maternal history of other psychiatric diagnosis; entitlement to special reimbursement for chronic disease (ever); preterm birth; neonatal care unit | SSRI exposure during pregnancy  AHR: 0.88 (0.65–1.20) |
| Viktorin et al. (2017) | The Swedish Medical Birth  Register | Exposed 3,982  Unexposed 172,646 | Birthdate; maternal and paternal age; father’s psychotropic medication that overlapped the pregnancy; mother’s one-time dispensations of psychotropic medication that overlapped the pregnancy; any diagnosis of specific psychiatric disorder sub-groups in either the mother and/or father’s life time | AD exposure during pregnancy  ARR: 1.23 (0.96–1.57) |
| Rai et al. (2017) | Stockholm youth cohort | Exposed 3,342  Unexposed 238,943 | Birth year; maternal psychiatric disorders diagnosed before birth; maternal medications used during pregnancy; sex; maternal age; paternal age; parity; maternal education; family income; maternal birth country | AD exposure during pregnancy  AOR: 1.45 (1.13–1.85) |
| Brown et al. (2017) | Health administrative data from Ontario, Canada | Exposed 2,837  Unexposed 33,069 | Top 500 covariates | SSRI exposure during pregnancy  AHR: 1.61 (0.997-2.59) |
| Sujan et al. (2017) | The Multi-Generation Register; the Prescribed Drug Register; the Medical Birth Register; the National Patient Register; the National Crime Register; the Swedish Register of Education | Exposed 1,574  Unexposed 1,558,085 | Parity, year of birth, paternal country of birth, age at childbearing, highest level of completed education, history of any criminal convictions, history of severe psychiatric illness, history of any suicide attempts, and maternal age at childbearing | AD exposure during first trimester  AHR: 0.83 (0.62-1.13)  SSRI exposure during first trimester  AHR: 0.81 (0.58-1.14) |
| Boukhris et al.  (2016) | Québec Pregnancy/Children Cohort. Régiede l’assurance maladie du Québec, Québec centralized hospitalization archives databases, Public Prescription Drug Insurance database of Québec, Québec Statistics database | Exposed:1,583  Unexposed:142,924 | The infant’s sex, year of birth, maternal age at first day of gestation, marital status, education level, social benefit recipient status, maternal psychiatric disorders in the year before or during pregnancy, chronic or gestational diabetes, and chronic or gestational hypertension | AD exposure during first trimester  AHR: 0.84 (0.52–1.36)  AD exposure during second and third trimesters  AHR: 1.75 (1.03–2.97) |
| Sorensen et al. (2013) | Danish Medical Birth Registry; Danish National Prescription Registry; Danish Psychiatric Central Register; Danish Civil Registration System; Danish National Hospital Register | ADs group: 104 with ASD; 8729 without ASD  Control group: 5333 with ASD; 641,449 without ASD | Maternal age at conception; paternal age at conception; parental psychiatric history (except maternal affective disorder); gestational age; birth weight; sex; parity | AD exposure during pregnancy  AHR: 1.5 (1.2–1,9)  SSRI exposure during pregnancy  AHR: 1.5 (1.2–1,9) |
| Hviid et al. (2013) | Danish Medical Birth Registry; Danish National Prescription Registry; Danish Psychiatric Central Register; Danish Civil Registration System | SSRIs group: 52 with ASD; 6016 without ASD  Control group: 3752 with ASD; 617,055 without ASD | Age; calendar period; mother’s age at birth; country of origin; place of residence; parity; psychiatric diagnoses before delivery; other drug use during pregnancy; smoking status; employment status; level of education | SSRI exposure during pregnancy  ARR: 1.20 (0.90–1.61) |
| Case-control Studies | | | | |
| Castro et al.  (2016) | The Partners HealthCare system; the Beth Israel Deaconess Medical Center; and the Boston Children’s Hospital | Cases: 1,245  Controls: 3,405 | Sex, race/ethnicity, year of birth, maternal insurance, maternal income, maternal major depressive disorder, and proxies for severity of illness | AD exposure during pregnancy  AOR: 0.90 (0.50-1.54) |
| Clements et al. (2015) | Partners HealthCare electronic health records | Cases: 1,377  Controls: 4,022 | Sex, race/ethnicity, birth year, insurance type, maternal age, and median income tertile + maternal major depressive disorder | AD exposure during pregnancy  AOR: 1.10 (0.70-1.70) |
| Gidaya et al.  (2014) | Danish Civil Registration System, Danish National Hospital Register, Danish Psychiatric Central Register, Danish Drug Prescription Register | Cases: 5,212  Controls: 52,150 | Parental age, child’s sex, history of maternal depression, other SSRI indications, and child’s date of birth (month and year) | AD exposure during pregnancy  AOR: 1.8 (1.4-2.3) |
| Harrington et al.  (2014) | Childhood Autism Risks from Genetics and the Environment Study, a standardized telephone interview with the child’s biological mother | Cases: 492  Controls: 320 | Center of birth, year of birth, and mother’s birthplace | AD exposure during pregnancy  AOR: 1.55 (0.59-4.08) |
| Rai et al.  (2013) | Stockholm Youth Cohort, Swedish National and Regional Register, Stockholm County Adult Psychiatric Outpatient Register, Swedish medical birth register | Cases: 1,679  Controls: 16,845 | Mother’s age, maternal psychiatric disorder, paternal age, parental income, education, occupation, mother’s birth country, and birth parity | AD exposure during pregnancy  AOR: 1.90 (1.15-3.14)  SSRI exposure during pregnancy  AOR: 1.65 (0.9-3.03) |
| Croen et al.  (2011) | Kaiser Permanente Medical Care Program in Northern California, Childhood Autism Perinatal Study, Pharmacy Information Management  System | Cases: 298  Controls: 1,507 | Age, race/ethnicity, maternal education, birth weight, sex, child’s year of birth, and place of birth | AD exposure during pregnancy  AOR: 2.0 (1.2-3.6)  SSRI exposure during pregnancy  AOR: 2.6 (1.3-5.4) |

Abbreviations: AD = Antidepressant; AHR = Adjusted hazard ratio; ARR = Adjusted relative risk; AOR = Adjusted odds ratio; ASD = Autism spectrum disorder; SSRI = Selective serotonin reuptake inhibitor
