# Supplementary material for: Association between maternal antidepressant use during pregnancy and autism spectrum disorder: an updated meta-analysis
Source: Mol Autism. 2018 Mar 27;9:21. doi: 10.1186/s13229-018-0207-7 (PMC5870683; doi:10.1186/s13229-018-0207-7)
Supplement: Supplementary file 3 — Table S2. Quality assessment of the included studies by the improved Newcastle–Ottawa Scale. (DOCX 16 kb) [file 13229_2018_207_MOESM3_ESM.docx]

| **Study** | **Design** | **Selection** | **Comparability** | | | **Exposure/Outcome** | | **Total scores** | |
| --- | --- | --- | --- | --- | --- | --- | --- | --- | --- |
| Malm et al. (2016) | **Cohort** | ★★★★ | | ★★ | ★★★ | | **9** | |  |
| Viktorin et al. (2017) | **Cohort** | ★★★★ | | ★★ | ★★ | | **8** | |  |
| Rai et al. (2017) | **Cohort** | ★★★★ | | ★★ | ★★ | | **8** | |  |
| Brown et al. (2017) | **Cohort** | ★★★★ | | ★★ | ★★★ | | **9** | |  |
| Sujan et al. (2017) | **Cohort** | ★★★★ | | ★★ | ★★★ | | **9** | |  |
| Boukhris et al. (2016) | **Cohort** | ★★★★ | | ★★ | ★★ | | **8** | |  |
| Sorensen et al. (2013) | **Cohort** | ★★★★ | | ★★ | ★★★ | | **9** | |  |
| Hviid et al. (2013) | **Cohort** | ★★★★ | | ★★ | ★★★ | | **9** | |  |
| Castro et al. (2016) | **Case-control** | ★★★ | | ★★ | ★★★ | | **8** | |  |
| Clements et al. (2015) | **Case-control** | ★★★★ | | ★★ | ★★★ | | **9** | |  |
| Gidaya et al. (2014) | **Case-control** | ★★★ | | ★★ | ★★★ | | **8** | |  |
| Harrington et al. (2014) | **Case-control** | ★★★★ | | ★★ | ★ | | **7** | |  |
| Rai et al. (2013) | **Case-control** | ★★★★ | | ★★ | ★★ | | **8** | |  |
| Coren et al. (2011) | **Case-control** | ★★★ | | ★★ | ★★★ | | **8** | |  |

Table S2. Quality assessment of the included studies by the improved Newcastle–Ottawa Scale
